# Retraction: miR-23a Targets Interferon Regulatory Factor 1 and Modulates Cellular Proliferation and Paclitaxel-Induced Apoptosis in Gastric Adenocarcinoma Cells

**DOI:** 10.1371/journal.pone.0234093

**Published:** 2020-05-29

**Authors:** The PLOS ONE Editors

After publication of this article [1], concerns were raised about the image data reported in Figs 1, 3, 5, and 7.

Concerns were raised about the reliability of colony formation assay results reported in Figs 1C, 5D, and 7C, in light of image similarities in the published figures. These concerns were not fully resolved by the materials provided post-publication in support of these figures.

- In Fig 1C, the plates representing MGC803/pcDNA3 and MGC803/pri-miR-23a appear similar to plates representing MGC803/ASO-23a and MGC803/ASO-NC, respectively.
- The Fig 1C, BGC823/pcDNA3 image appears similar to the Fig 5D, BGC823/IRF1 panel, when rotated 90 degrees.
- pcDNA3 and pri-miR-23a plates shown for MGC803 in Fig 1C appear similar to the pcDNA3 and pri-miR-23a/pcDNA3 plates shown for MGC803 in Fig 7C, respectively.
- The plate shown in Fig 1C for BGC823/ASO-23a appears similar to the plate shown in Fig 7C for BGC823/pcDNA3.

In each case the similar images represent different experiments. The authors noted that errors were made in preparing the figures resulting in duplication of the above images. The authors also noted that Fig 5C was correct, and that the correct Fig 1 MGC803/ASO-23a image was erroneously included in the published Fig 7 as representing MGC803/pri-miR-23a/IRF1.

Updated versions of Figs 1 and 7 were provided in response to the above issues. In the updated version of Fig 7C, three of the MGC803 images appear similar to images reported in Fig 5D as representing results of different experiments.

In addition, concerns were raised about western blot data reported in Figs 3E, 5B, and 7A.

- In Fig 3E, there appear to be vertical discontinuities after lanes 1, 2 of the MGC803/IRF1 blot, and after lane 2 of the BGC823/IRF1 blot. The authors confirmed that different images were spliced in preparing this figure. They provided original image data for MGC803 results, and replicate data for the BGC823 experiment. Raw image data underlying Fig 3E BGC823 blots were not provided. Due to data labeling discrepancies and other issues involving the files provided post-publication, there remain unresolved concerns about the reliability of the results reported in this figure.
- Lanes 3–4 of the MGC803 GAPDH panel in Fig 5B appear similar to lanes 3–4 of the BGC823 IRF1 panel in Fig 3E. Also, in Fig 5B, lane 4 of the MGC803 GAPDH blot appears similar to lane 1 of the BGC823 GAPDH blot. According to data provided for Fig 5B, the GAPDH results shown in this published figure were not the matched controls for the corresponding IRF1 data. The BGC823 GAPDH blot data provided for Fig 5B appeared to match the BGC823 GAPDH data reported in Fig 3, where different IRF1 results were reported.
- In Fig 5B, there appear to be vertical discontinuities after lanes 2, 3 of the MGC803 IRF1 blot and after lane 2 of the BGC823 IRF1 blot. The authors provided raw image data supporting the Fig 5B panels. The raw data confirm that lanes were rearranged in preparing Fig 5B, and that the pSilencer and sh-IRF1 data in the published figure for BGC823 originate from a different blot than that which served as the source of the other IRF1 data in the figure.
- Lanes 2–4 of the BGC823 GAPDH panel in Fig 5B appear similar to lanes 1–3 of the BGC823 GAPDH panel in Fig 7A. The corresponding lanes represent different experiments in the two figures. The authors provided supporting data which indicated that incorrect GAPDH were reported in both figures.

The *PLOS ONE* Editors have determined that the reliability of results reported in this article is in question given the extent of figure errors in the published article and concerns that arose during our review of supporting files provided post-publication. Therefore, the *PLOS ONE* Editors retract this article.

XL, JR, JZ, ML, and HT agreed with retraction and apologized for the issues with the published article. L-hZ and XL either could not be reached or did not reply directly.
